# Both Size and GC-Content of Minimal Introns Are Selected in Human Populations

**DOI:** 10.1371/journal.pone.0017945

**Published:** 2011-03-17

**Authors:** Dapeng Wang, Jun Yu

**Affiliations:** 1 CAS Key Laboratory of Genome Sciences and Information, Beijing Institute of Genomics, Chinese Academy of Sciences, Beijing, People's Republic of China; 2 Graduate University of Chinese Academy of Sciences, Beijing, People's Republic of China; Aarhus University, Denmark

## Abstract

**Background:**

We previously have studied the insertion and deletion polymorphism by sequencing no more than one hundred introns in a mixed human population and found that the minimal introns tended to maintain length at an optimal size. Here we analyzed re-sequenced 179 individual genomes (from African, European, and Asian populations) from the data released by the 1000 Genome Project to study the size dynamics of minimal introns.

**Principal Findings:**

We not only confirmed that minimal introns in human populations are selected but also found two major effects in minimal intron evolution: (i) Size-effect: minimal introns longer than an optimal size (87 nt) tend to have a higher ratio of deletion to insertion than those that are shorter than the optimal size; (ii) GC-effect: minimal introns with lower GC content tend to be more frequently deleted than those with higher GC content. The GC-effect results in a higher GC content in minimal introns than their flanking exons as opposed to larger introns (≥125 nt) that always have a lower GC content than that of their flanking exons. We also observed that the two effects are distinguishable but not completely separable within and between populations.

**Conclusions:**

We validated the unique mutation dynamics of minimal introns in keeping their near-optimal size and GC content, and our observations suggest potentially important functions of human minimal introns in transcript processing and gene regulation.

## Introduction

Intron is an important and necessary subassembly of eukaryotic genes and plays precise and complicated roles especially in the case of mRNA processing and alternative splicing [1], [2], [3], [4]. Although introns are not considered as at the same importance level as protein-coding sequences, increasing evidence has revealed potential function-related selections on introns and their sequences [5], [6], [7], [8]. Debates on intron gain and loss as well as other evolutionary features are still ongoing [9], [10], [11], [12]. In an early study, we defined a size optimum (∼100 bp) in the distribution of all human introns and named these introns as “minimal introns” [13]. We also discovered an effect that the length of minimal introns tended to be maintained at an optimal size based on re-sequencing 93 representative minimal introns and 12 indels in an average of 45.7 random samples from a world-wide human population [13]. We further analyzed human minimal introns and minimal intron-containing genes (MIGs), and showed many their unique features, including non-random distribution among human chromosomes, tendency to reside near the 3′ end of transcripts, and higher expression level than other genes [14].

There have been several comparative studies within and among species, revealing situations where short introns (or minimal introns) are under selection, and most of them have focused on model organisms, such as Drosophila. There have been several interesting observations. First, shorter introns tend to have higher recombination rates [15]. Second, there are more deletions than insertions occurring among all intronic regions, but very short introns counterwork the strength of deletions [15], [16], [17], [18], [19]. Third, deleterious shorter deletions and rare larger insertions may be fixed by drift and selection in the process of speciation and population differentiation [16]. Fourth, shorter introns at different positions of the same gene tend to suffer different selective constraints [16]. Fifth, intron GC content and sequence divergence tend to have an adverse correlation [20]. Last, adaptive and purifying selection can work together to keep the lengths of short introns around an optimum size [19]. However, these observations are not yet exploited in vertebrate and human populations.

As data from the 1000 Genome Project are being released [21], we have new opportunities to revisit some of the issues unresolved in the past due to limited resource and inadequate data. From a recent release of 179 re-sequenced human genomes, limited to three populations of 59 YRI (Africans), 60 CEU (Europeans), and 60 JPTCHB (Asians) individuals, we obtained 265, 145, and 188 indels in minimal introns (50–124 nt). Our results demonstrated that minimal introns have selective constraint in human populations, which is defined as two distinct yet interrelated effects—size and GC effects.

## Results

### Key parameters

**Figure 1:**
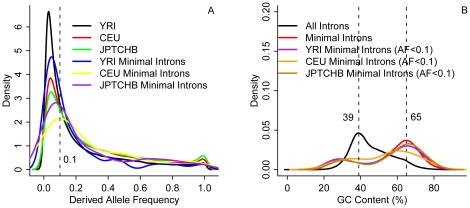
Estimation of key parameters. The distributions of derived allele frequency or DAF (A) and GC content (B) of minimal introns among the three studied populations: Africans (YRI), Europeans (CEU), and Asians (JPTCHB). The dashed lines divide the thresholds for DAF (A) and intron GC content (B).

To obtain reliable sequence and allele information, we limited our dataset to (1) ancestor alleles were determined based on sequences from all three primates and (2) intron sequences must be mapped precisely in genomic sequences. This filter yielded 202,009 YRI, 146,842 CEU, and 136,175 JPTCHB indels. When plotted the derived allele frequency (DAF) of the indel data, we found that all curves showed a uni-modal distribution, slightly skewed toward left (Figure 1A). The more ancient population, YRI, possessed more indels in lower frequencies than two other populations, CEU and JPTCHB; this result is in agreement with previous findings and the phenomenon was attributable to the bottleneck effect in the evolution of CEU and JPTCHB populations [22]. To balance between capturing more data and avoiding the influence of strong selection, we subsequently chose the frequency value corresponding to the peak on the rightmost as a cut-off and defined the indels with DAF<0.1 as the less-selection variations. We had 55.09% YRI, 31.03% CEU, and 39.89% JPTCHB indel data when the percentage of minimal introns under the condition of DAF<0.1 over all examined minimal introns (Figure 1A). We also plotted the distribution of GC contents of the selected minimal introns (DAF<0.1) in comparison to those of total introns and total minimal introns (Figure 1B). We discovered that the GC content in all introns peaked at 39% but that the rest of the curves related to minimal introns peaked at 65%. Obviously, minimal introns tended to have higher GC content than larger introns. Therefore, we considered 65% as the default cut-off to study GC content effect on minimal intron evolution.

### The size-effect: the RDI of minimal introns with a length range of 88–124 nt is higher than those with a shorter length range of 50–86 nt

**Figure 2:**
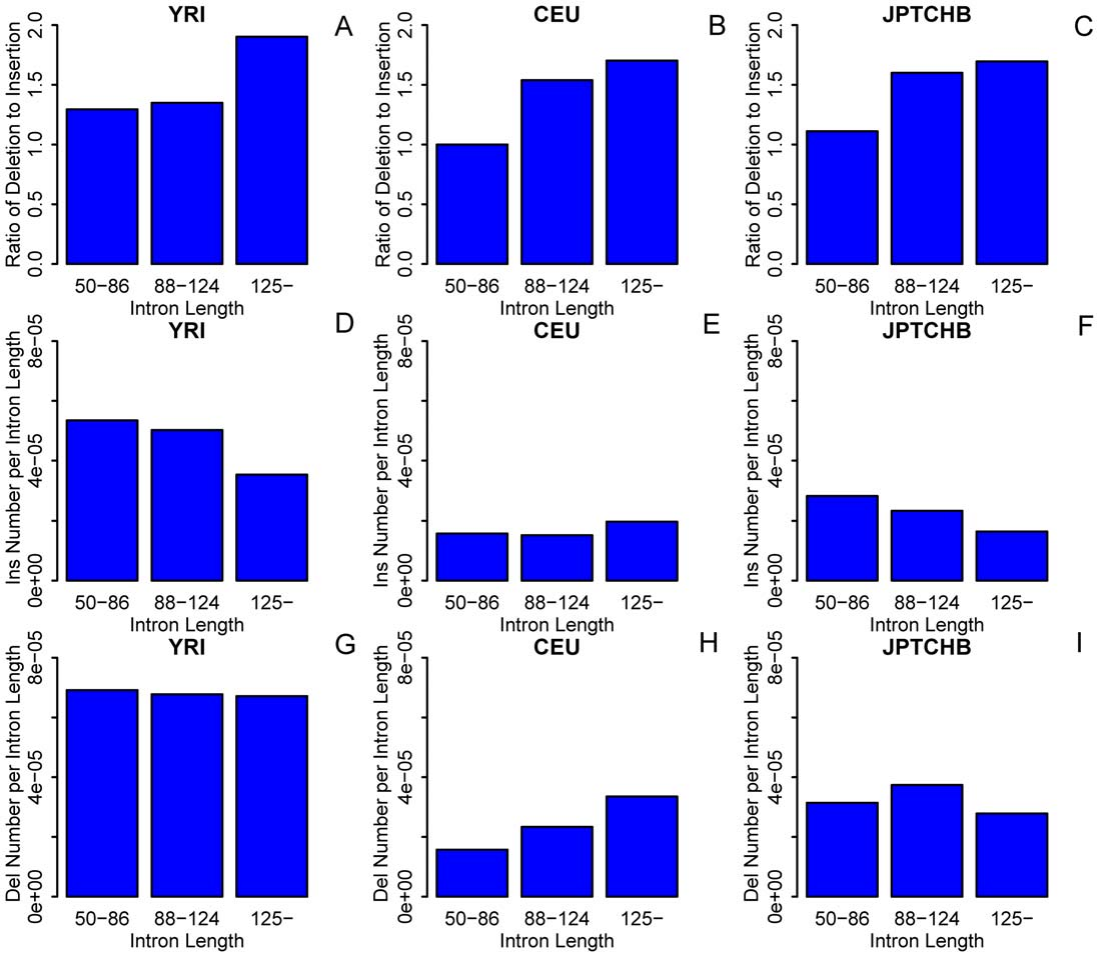
Indel counts and ratios. RDI (A, B, C), insertions (D, E, F) and deletions per intron length (G, H, I) in different minimal intron size intervals of the three populations (DAF <0.1).

To study whether there is an optimal size for minimal introns, we defined 87 nt as an *optimal value* based on the intron length distribution corresponding to the peak value (mode) of minimal introns [14]. The reason why we defined the introns in a length range of 50–124 nt as minimal introns is that the two extremes of minimal introns have the same distance away from 87 nt and this range provides a reasonable baseline for examining indel variations in the two intervals of 50–86 nt and 88–124 nt. We also analysed the larger introns (≥125 nt) as controls. In particular, we developed a notation to differentiate the preference of deletion over insertion: *ratio of deletion to insertion* or RDI when we investigated strength variations around an optimum: 87 nt (Figure 2A–C). We made several interesting observations: (i) The RDIs are always greater than one in all intron length intervals in samples from all three populations. (ii) RDI increases with an increasing intron length and larger introns tend to have more deletions than shorter introns, perhaps to protect intron from over expansion. (iii) There were higher RDIs in those minimal introns with a size range of 88–124 nt than the narrower-ranged ones (50–86 nt), and the higher RDI suggested that minimal introns have a tendency to maintain an optimal size around 87 nt. In other words, taken the consideration that the tendency to delete is characteristic to all introns, a lower RDI suggested biased insertion trend, which actually happens in the minimal introns with a length range of 50–86 nt.

We also strengthened this conclusion by examining RDI in combined dataset of the three populations and by examining other intron length intervals (Tables 1, 2). To interrogate the occurrence of insertion event, we also plotted insertion events normalized over all intron lengths for different intervals; as a control, we normalized deletion events normalized over all intron lengths for different intervals (Figure 2D–I). Since our data used in this study are all from autosomal regions, we only adopted intron data in euchromosomes for computing the parameters related to indel events per intron length. There is a decreasing trend of insertion events per intron length as intron length increases in both YRI and JPTCHB (Figure 2D and F). Although insertion event per intron length of minimal introns is smaller than that of larger introns, the insertion events per length of 50–86 nt is greater than that of introns with a length range of 88–124 nt in CEU (Figure 2E). All these results support the observation that there are more insertion events happening in minimal introns with lengths <87 nt than those with lengths >87 nt. This phenomenon may also be attributable to a protective selection that shorter introns are deleterious for the splicing machinery [23], [24]. Furthermore, we found a deletion bias in those minimal introns >87 nt when compared to those <87 nt in two populations of the three populations, CEU and JPTCHB (Figure 2H and I). We also did an analysis using the combined dataset from the three populations, yielding: (1) the value of deletion event per intron length for the minimal introns >87 nt in size was 1.051E−04, which was greater than that of 1.038E−04 for those <87 nt in size; (2) the value of insertion event per intron length for the minimal introns <87 nt in size was 9.432E−05, which was greater than that of 8.411E−05 for those >87 nt in size. In conclusion, minimal introns <87 nt in size are more likely to have insertion mutations, whereas those >87 nt in size are more likely to delete sequences. This validated our previous discovery that minimal introns have a tendency to maintain an optimal size in a population or evolutionary process. We attributed this as the size-effect.

**Table 1:** RDI (DAF<0.1) in different intron size intervals from combined datasets of the three populations.

	Length		GC Content	
Intron Length	<87 nt	>87 nt	<65%	>65%
50 nt–124 nt	1.100 (33/30)	1.250 (90/72)	1.534 (89/58)	0.870 (40/46)
70 nt–104 nt	1.103 (32/29)	1.256 (49/39)	1.903 (59/31)	0.718 (28/39)
50 nt–150 nt	1.100 (33/30)	1.353 (138/102)	1.697 (129/76)	0.828 (48/58)

**Table 2:** RDI (DAF<0.1) in different intron size intervals of the three populations.

	YRI		CEU		JPTCHB	
Intron Length	<87 nt	>87 nt	<87 nt	>87 nt	<87 nt	>87 nt
70 nt–104 nt	1.313	1.400	1.000	1.250	1.111	1.692
54 nt–120 nt	1.294	1.459	1.000	1.900	1.111	1.550
50 nt–140 nt	1.294	1.315	1.000	1.813	1.111	1.333
50 nt–150 nt	1.294	1.311	1.000	2.118	1.111	1.441

### The GC-effect: RDI of minimal introns with GC<65% is higher than those with GC>65%

**Figure 3:**
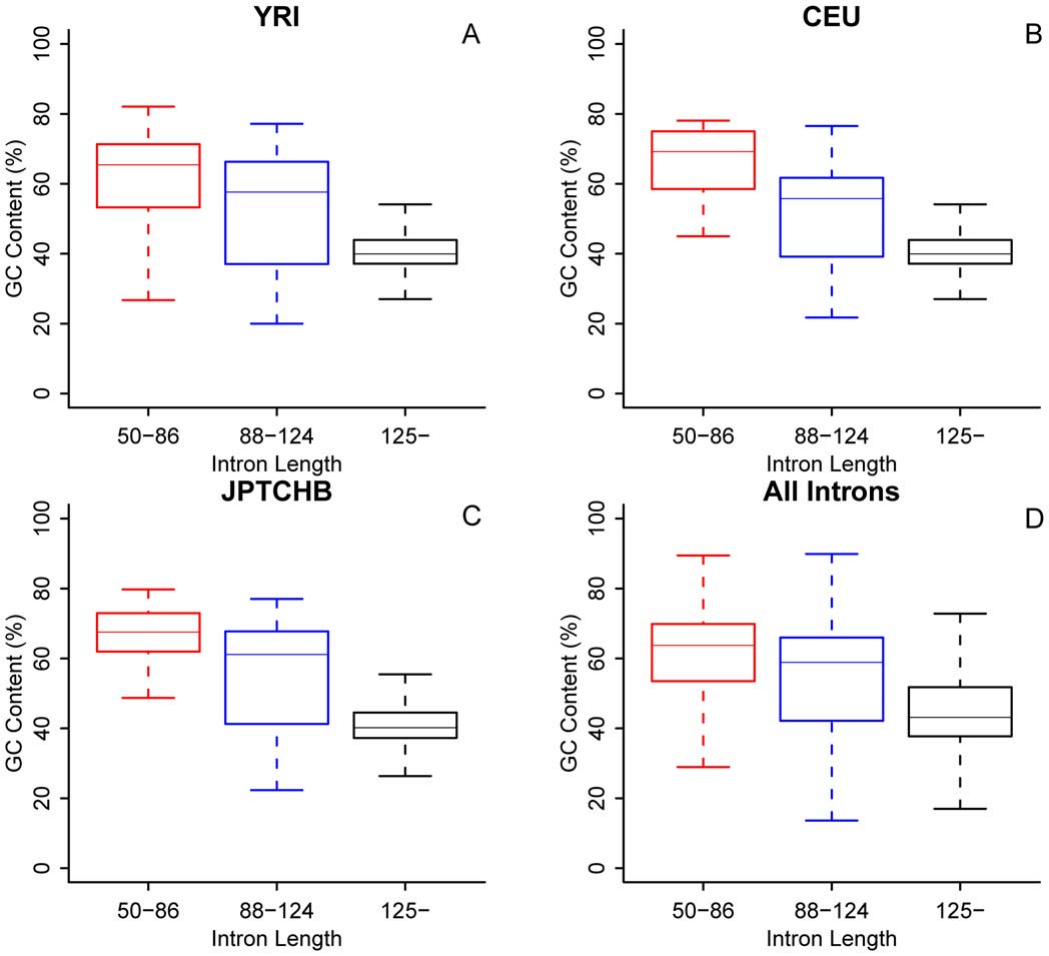
Relationship between GC content and intron size. GC contents in three size intervals from the three populations (DAF <0.1): YRI (A), CEU (B), and JPTCHB (C) as well as the combined data (all introns; D). The horizontal lines in the boxplots indicate the upper, the median, and the lower quartiles (from the top to the bottom).

GC content is an important dimension of DNA compositional dynamics related to gene and genome evolution [25]. We investigated how GC content influences RDI by separating minimal introns into two portions at 65%. We found that the RDI of minimal introns with GC contents <65% is greater than those with GC contents >65% regardless population variations, i.e. minimal introns with lower GC contents are more likely to be deleted than those with higher GC contents (Tables 1, 3). When further studied the combined dataset of three populations, we found that the deletion event normalized by intron length was 1.098E−04 for minimal introns with GC<65% was larger than that of those with GC>65%, 1.018E−04; similarly, the insertion event normalized by intron length for the two minimal intron categories were 1.171E−04 for those GC>65% and 7.157E−05 for those GC<65%. We further examined this GC-effect in three different intron size intervals in populations (Figure 3) and confirmed that the shorter the intron length, the higher the GC content. To explore the reason why minimal introns tend to have a high GC content, we computed the GC contents for their 5′- and 3′-flanking exons (Table 4). We found that minimal introns overall have significantly higher GC-contents than their flanking exons as opposed to those of larger introns. Finally, when relating GC-effect to size-effect, we found that shorter introns (50–86 nt) with higher GC content tend to harbor more insertions than longer introns (87–124 nt) with lower GC content. Furthermore, our polymorphism data supported the idea that insertions in shorter introns with higher GC content lead to intron expansion with decreased GC content, while deletions in longer introns with lower GC content result in intron contraction with increased GC content. The GC content of minimal introns therefore tends to reach their optimal length and higher content than their flanking exons.

**Table 3:** RDI (DAF<0.1) under variable size intervals and GC-contents.

	YRI		CEU		JPTCHB	
Intron Length	<65%	>65%	<65%	>65%	<65%	>65%
50 nt–124 nt	1.686	0.926	1.667	1.167	2.071	1.000
70 nt–104 nt	2.600	0.609	1.375	1.200	2.333	1.000
50 nt–150 nt	1.638	0.879	2.571	0.875	1.909	0.909

**Table 4:** Median GC contents in variable intron size intervals and regions.

Intron Length	5′-Exon	Intron	3′-Exon	P-Value*
50 nt–86 nt	59.44%	63.53%	59.26%	<2.2e−16
88 nt–124 nt	56.41%	58.82%	55.82%	<2.2e−16
125 nt -	50.34%	43.07%	48.65%	<2.2e−16

*We pooled both 5′-exon and 3′-exon data as flanking exon data and compared them with intron data. The *P* values were calculated based on Wilcoxon rank sum test.

### Are size-effect and GC-effect mutually independent?

We performed further analyses with a combined dataset from the three populations to distinguish the relationship between GC-effect and size-effect. We first separated all minimal introns into two groups based on their relative lengths around 87 nt and used this grouping scheme to eliminate the intron size-effect. We subsequently examined the GC-effect between the groups and chose an appropriate GC content cut-off to catch a comparable number of indels in the two intervals (Table 5). We found that under all conditions, including three different definitions of minimal intron lengths, the GC-effect does exist. Furthermore, when considering the three populations separately, we found that the GC-effect hold in two out the three populations, YRI and JPTCHB, but became obscure in CEU (data not shown). There are two possible reasons to explain the results: there were not enough data in CEU or the GC-effect is dependent on the size-effect in certain populations. When examining the dependence of size-effect on GC-effect, we observed that a majority of RDIs appeared against size-effect found in every minimal intron intervals we defined after removing the impact of GC-effect (Table 6). Therefore, we believe that the size-effect and GC-effect are distinguishable to a certain extent but may not be separable completely since some of the GC-rich minimal introns may also be a result of weaker purifying selection in size but strong positive selection in GC content. The higher GC content among minimal introns adds a significant footnote to this reasoning.

**Table 5:** RDI (DAF<0.1) when examining dependence of GC-effect on intron length.

	<87 nt		>87 nt	
Intron Length	GC%	RDI	GC%	RDI
50 nt–124 nt	<67.1%	2.100 (21/10)	<57.7%	1.314 (46/35)
	>67.1%	0.600 (12/20)	>57.7%	1.189 (44/37)
70 nt–104 nt	<67.0%	2.000 (20/10)	<59.0%	1.316 (25/19)
	>67.0%	0.632 (12/19)	>59.0%	1.200 (24/20)
50 nt–150 nt	<67.1%	2.100 (21/10)	<57.6%	1.667 (75/45)
	>67.1%	0.600 (12/20)	>57.6%	1.105 (63/57)

**Table 6:** RDI (DAF<0.1) when examining dependence of size-effect on GC content.

Intron Length	Intron Interval	GC%<65%	GC%>65%
50 nt–124 nt	<87 nt	2.111 (19/9)*	0.667 (14/21)
	>87 nt	1.327 (65/49)	1.087 (25/23)
70 nt–104 nt	<87 nt	2.111 (19/9)*	0.650 (13/20)
	>87 nt	1.591 (35/22)	0.824 (14/17)
50 nt–150 nt	<87 nt	2.111 (19/9)*	0.667 (14/21)
	>87 nt	1.567 (105/67)	0.943 (33/35)

*These data points are not in agreement with the size-effect.

### Other evolutionary parameters

To see if selective sweeps on coding and intron regions from the same genes play any roles in minimal intron selection, we examined Ka/Ks values of the two flanking coding exons of minimal introns of MIGs in the length intervals of 50–86 nt and 88–124 nt from the combined dataset, and found that the median selective pressure of the flanking coding exons of MIGs with intron length of 50–86 nt were slightly smaller than that with intron length of 88–124 nt (0.037 vs 0.062, P-value = 0.1175; Wilcoxon rank sum test). The results suggested that shorter introns are subjected to stronger selective constraints than longer introns when we defined the length of 87 nt as an optimum. We believe that the insignificance of the test is largely due to insufficient data. The stronger negative selection acting on the nucleotide content of protein coding sequences provides additional evidence to support the proposal that minimal introns in a size range of 50–86 nt are more likely to subject to purifying selection resisting to the overall deletion force acting in all introns.

## Discussion

In the current study, our samples are still limited to the three populations and limited number of indels in minimal introns (50–124 nt). Although the statistics based on the current data is not as striking as the previous result, they are more robust and conclusive. In the previous study, we chose a set of introns that cover a full range of intron GC content (16.4–78.7%) in order to avoid biases in sampling. As a matter of fact, the GC content of minimal introns in the human genome tends to be higher (median: 60.4%), and in a rather random sampling scheme, our analysis is biased toward minimal introns with higher GC content whereas in a more selective case we actually sampled more AT-rich minimal introns. In either case, size-effect is indisputable. In the previous study, by using low-frequency alleles (indels with DAF<0.06), we found the size-effect but failed when look at higher frequencies, such DAF>0.35. Similarly, in this study we also tried to examine various DAF cut-offs (e.g. 0.2, 0.3 and 0.4) and found that the size-effect became weaker when DAF cut-off is above 0.1 (data not shown). Our data collectively confirmed the proposal that minimal introns have an optimal size or a narrow range and the distribution of minimal introns has a mode of 87 nt. If introns drift off this optimal size, there should be a tendency to be drawn back. To look for evidence supporting our hypothesis, we chose to focus on minor alleles that are sensitive to selection but not yet fixed in populations as we have preciously pointed out that rare polymorphisms (as a threshold of 0.06) are subjected to selections (e.g. adaptive selection on protein-coding sequences) [26]. Our current study has taken advantage of this phenomenon and it is why rare indels worked well for discovering size-effect. In addition, the result from our current study excluded population biases since YRI, CEU and JPTCHB are relative purer populations as compared to what used in our previous work.

In the current study, we also addressed GC-effect in addition to size-effect. In the previous study, we noticed a GC-effect where almost all insertions happened in GC-rich minimal introns whereas all deletions are among GC-poor minimal introns. Therefore, our previous data were not informative when we tried to separate size effect from GC effect. Although the current data did not give us a clear-cut answer but we believe that GC-effect should be resolved with statistical significance based on adequate data from a broad GC-content range.

Finally, we would like to emphasize that sequence variations represent a rich data resource for interrogating biological questions, especially in a context of structure-function relationship such as the size of minimal introns and exons. In a long enough generation period, indels (or SNPs) with higher derived allele frequencies are more likely to be related to positive selections (adaptive selections), as those with lower derived allele frequencies tend to be under negative selections (purifying selections) for deleterious variants, in particular, the extremely rare variations. In contrast, what we found is that the DAF<0.1 variations appear largely adaptive in human populations because the maintenance of an optimal minimal intron length results a better functionality (efficiently processed and exported to the cytosol) for minimal intron-containing genes. We considered the indels of minimal introns as a dynamic parameter for reaching the size optima under various selective forces. Both positive and negative selections can work on these indels; positive selection leads to the fixation of the minimal introns with optimal sizes and purifying selection tends to remove the minimal intron-containing genes (or the individuals in a population) that possessing over- or under-sized minimal introns.

Parsch et al. observed the insertion bias in short introns when examining indel divergence between or within *Drosophila melanogaster* [19]. They also found that the occurrence of insertions may reflect the effect of selection by increasing their frequencies. At the same time, a deletion bias was found in short introns in the lineage of *D. simulans* [19], [27]. Both results supported a “selective force” model: indels in the short introns are more likely to be fixed by selection or by genetic drift. In contrast, our results may provide evidence for an alternative “mutational force” model: introns deviate from a length optimum tends to have indels. We proposed that the two models may work in different species, such as insects and vertebrates. More detailed studies in short introns from large number of species need to be done in the future. Our work emphasized the potential functional roles for minimal introns in the lineages of vertebrates, and provided a reliable theoretical basis for the future function validation both experimentally and *in silico*.

## Materials and Methods

We defined intron locations based on human Refseq data (NCBI build 36) described previously [14]. In general, we considered introns in a length range of 50–124 nt as minimal. We collected indel data (produced based on Dindel protocol [28]) from the 1000 genome database and three populations (YRI, CEU and JPTCHB) [21] (ftp://ftp-trace.ncbi.nih.gov/1000genomes/). We retrieved the three blastz alignment files from UCSC databases [29] (ftp://hgdownload.cse.ucsc.edu/) between human and another species: chimpanzee (hg18vsPanTro2), orangutan (hg18vsPonAbe2), and rhesus monkeys (hg18vsRheMac2). We compared the indel variations with the corresponding position of these three primate references to identify ancestral alleles. We calculated the nonsynonymous and synonymous substitution rates between human and mouse from *γ*-MYN method [30] using KaKs_Calculator2.0 [31] as evolutionary parameters of the flanking coding regions of minimal introns in minimal intron-containing genes. We examined all parameters under the condition of derived allele frequency <0.1 to avoid interference from strong positive selections on indels.
